# Evaluating Infertility Through Diagnostic Hysterolaparoscopy: A Prospective Study

**DOI:** 10.7759/cureus.76314

**Published:** 2024-12-24

**Authors:** Aparna Sarwade, Hemant G Deshpande, Shriraj Katakdhond, Saba Chaudhary

**Affiliations:** 1 Obstetrics and Gynaecology, Dr. D Y Patil Medical College, Hospital and Research Centre, Dr. D Y Patil Vidyapeeth (Deemed to be University) Pimpri, Pune, IND

**Keywords:** diagnostic hystero-laparoscopy, infertility, ovarian factors, reproductive health, tubal factors

## Abstract

Background

Infertility affects a significant proportion of reproductive-age couples globally, with diverse causes. Diagnostic hystero-laparoscopy (DHL) is emerging as a preferred diagnostic tool for evaluating infertility, combining laparoscopy and hysteroscopy for comprehensive assessment.

Objective

The primary objective of this prospective study was to evaluate the diagnostic accuracy and clinical utility of DHL in identifying the causes of infertility in women. Secondary objectives included assessing complication rates associated with the procedure and comparing the efficacy of DHL with other diagnostic modalities to determine its relative benefits and safety in clinical practice.

Methods

Fifty female patients (ages 20-40 years) experiencing primary or secondary infertility were enrolled from the Outpatient Department of the tertiary care center. Patients were selected based on specific inclusion and exclusion criteria. Informed consent was obtained, and detailed medical histories were recorded. All participants underwent DHL, assessing uterine, tubal, and ovarian factors contributing to infertility.

Results

Of the 50 patients, 36 (72%) had primary infertility and 14 (28%) had secondary infertility. The predominant age group for primary infertility was 26-30 years (16 patients, 44.4%), while secondary infertility was most common in the 31-35 age group (7 patients, 50%). Tubal factors accounted for 18 cases (36%), while ovarian issues were identified in 15 cases (29.8%). Uterine factors were found in five cases (11.1% for primary, 7.14% for secondary). DHL also identified uterine pathologies that may lead to pregnancy complications.

Conclusions

The study underscores the multifactorial nature of infertility, highlighting the importance of DHL in its evaluation. The findings advocate for advanced diagnostic techniques to facilitate targeted treatments and enhance reproductive outcomes.

## Introduction

Infertility is a complex health issue with significant social and economic implications, affecting 10-15% of reproductive-age couples globally [1]. It is defined as the inability to conceive after 12 months of regular, unprotected intercourse [2]. Infertility can be classified into two types: primary infertility, where conception has never occurred, and secondary infertility, where a couple is unable to conceive after a previous pregnancy [3,4]. The cause of infertility varies, with female factors contributing to 40-55% of cases, followed by male factors, which account for 30-40% [3-5]. Despite advances in medical understanding, approximately 10% of infertility cases remain unexplained.

Several factors contribute to the rising incidence of infertility, including lifestyle choices, career ambitions, environmental influences, and delayed marriage [6-8]. Traditional diagnostic methods, such as pelvic examinations, may not always detect the underlying issues, which has led to the adoption of more advanced diagnostic tools [9]. Diagnostic hystero-laparoscopy (DHL) has emerged as a gold standard in infertility assessment, combining laparoscopy and hysteroscopy [10,11]. Laparoscopy offers a panoramic and magnified view of the reproductive organs, aiding in both diagnosis and treatment, while hysteroscopy provides a safe and effective alternative for diagnosing and addressing intrauterine conditions. Together, these techniques significantly enhance the accuracy and therapeutic potential of infertility management.

## Materials and methods

Study design

This was a prospective study involving 50 female patients who were experiencing primary or secondary infertility. The study was carried out at the Outpatient Department (OPD) of D.Y. Patil Medical College, Hospital, and Research Center, Pune. The study population was drawn from patients attending the OPD for infertility-related concerns. The study was approved by the Institutional Ethical Committee (IESC/W/179/2024).

Participant selection

Patients were selected based on specific inclusion and exclusion criteria. Female patients between the ages of 20 and 40 years who were experiencing either primary infertility (inability to conceive despite regular, unprotected intercourse) or secondary infertility (inability to conceive after a previous pregnancy) were included. To ensure accurate results, patients with active pelvic inflammatory disease (PID), significant medical comorbidities, or those who were morbidly obese were excluded from the study. These conditions could have complicated the evaluation or increased the risk of complications during the diagnostic procedures.

Consent and data collection

Prior to data collection, informed consent was obtained from each participant, ensuring they were fully aware of the study’s purpose, procedures, and potential risks. Once consent was granted, a detailed medical history was recorded for each patient, covering factors such as menstrual history, sexual health, previous pregnancies, and any prior medical treatments. A thorough clinical examination was then performed to assess the patients' overall health and identify any obvious signs of reproductive abnormalities.

Diagnostic procedures

All patients underwent DHL, a minimally invasive procedure that combines both laparoscopy and hysteroscopy under general anesthesia. DHL was utilized to conduct a thorough evaluation of both uterine and tubal factors that could contribute to infertility. This procedure involved several key assessments. For uterine findings, the evaluation focused on determining the position of the uterus (whether anteverted or retroverted), assessing the size of the uterus (to identify if it was normal or hypoplastic), and identifying any abnormalities such as fibroids or polyps. Additionally, any structural anomalies of the uterus were also detected.

Regarding tubal findings, DHL provided insights into the patency and condition of the fallopian tubes. The assessment included checking whether the tubes were normal, or if there were unilateral or bilateral blockages. The evaluation also looked for the presence of hydrosalpinx, characterized by distention, thin or translucent walls, and fluid accumulation. Peritubal adhesions and the condition of the fimbriae were assessed to determine the extent of damage. Other observations included the identification of any hypoplastic (underdeveloped) tubes, tubular masses, or unilateral and bilateral cornual blockages (blockages at the points where the tubes enter the uterus).

For ovarian findings, the assessment focused on determining if the ovaries were normal or enlarged. The examination also looked for the presence of specific conditions such as streak ovaries, polycystic ovary syndrome (PCOS), and any cysts including ovarian, chocolate, or follicular cysts. Additionally, signs of ovulation were assessed to further understand the functional status of the ovaries. The ovaries were inspected for features of PCOS, including enlargement, a thickened white capsule, and multiple small cysts resembling a "string of pearls". Adhesions around the ovaries and fallopian tubes were identified and treated as required. In cases where ovulation induction was indicated, laparoscopic ovarian drilling was performed to enhance fertility.

Other factors considered during the DHL procedure included tubo-peritoneal conditions, which are crucial in understanding fertility issues. This involved checking for the presence of endometriosis, pelvic adhesions, and signs of tuberculosis. Each of these conditions can significantly impact reproductive health and contribute to infertility, making their evaluation an essential part of the comprehensive diagnostic approach.

Statistical analysis

Descriptive statistics were used to summarize the demographic and clinical characteristics of the study participants, including frequencies and percentages for categorical variables. For comparative analysis, Chi-square tests were utilized to analyze associations between categorical variables. A p-value of less than 0.05 was considered statistically significant. All statistical analyses were performed using GraphPad Prism 10 (La Jolla, CA, USA).

## Results

Out of 50 patients assessed in this study, 36 patients (72%) were diagnosed with primary infertility, while 14 patients (28%) had secondary infertility. We assessed the age distribution of patients with primary and secondary infertility (Table 1). Among the 12 patients aged 21-25, 10 (27.7%) had primary infertility, while two (14.28%) had secondary infertility. In the 26-30 age group, which comprised 20 patients, 16 (44.4%) had primary infertility and four (28.57%) had secondary infertility. For patients aged 31-35, out of 15 individuals, eight (22.2%) had primary infertility, and seven (50%) had secondary infertility. Lastly, in the 36-40 age group, consisting of three patients, two (5.55%) had primary infertility, and one (7.14%) had secondary infertility. The Chi-square analysis for independence revealed that there was no significant association between the age and type of infertility.

**Table 1:** Distribution of patients with age and corresponding type of infertility P-value less than 0.05 is significant

Age (Years)	Primary n(%)	Secondary n(%)	Total	Chi-square Value	P-value
21-25	10(27.7%)	2(14.28)	12	4.029	0.258
26-30	16(44.4)	4(28.57)	20		
31-35	8(22.2)	7(50)	15		
36-40	2(5.55)	1(7.14)	3		
TOTAL	36(100%)	14(100%)	50		

In this study we further examined the duration of infertility among patients, categorizing them based on the number of years they had experienced infertility (Table 2). For those with primary infertility, 24 patients (66.6%) had been infertile for one to five years, while eight patients (22.2%) had infertility lasting six to 10 years. Only three patients (8.3%) had been infertile for 11-15 years, and one patient (2.7%) for 16-20 years. In contrast, among patients with secondary infertility, three individuals (21.4%) had experienced infertility for one to five years, eight patients (57.1%) had infertility lasting six to 10 years, two patients (14.2%) had been infertile for 11-15 years, and one patient (7.14%) for 16-20 years. The Chi-square test revealed a statistically significant association between the duration of infertility and infertility type (primary vs. secondary), with a Chi-square value of 8.5 and a p-value of 0.037.

**Table 2:** Distribution of patients based on the duration of infertility p-value less than 0.05 is significant

Duration ( Years)	Primary n(%)	Secondary n(%)	Total n(%)	Chi-square Value	P-value
1-5	24(66.6%)	3(21.4%)	27(54%)	8.5	0.037
6-10	8(22.2%)	8(57.1%)	8(57.1)		
11-15	3(8.3%)	2(14.2%)	2(14.2)		
16-20	1(2.7%)	1(7.14%)	1(7.14)		
TOTAL	36	14	50(100%)		

The present study also analyzed various factors contributing to infertility in patients with primary and secondary infertility (Table 3). Uterine factors were identified in four patients (11.1%) with primary infertility and one patient (7.14%) with secondary infertility, totaling five cases. Tubal issues were more prevalent, affecting 12 patients (33.3%) with primary infertility and six patients (42.85%) with secondary infertility, amounting to a total of 18 cases. Ovarian problems were observed in 11 patients (30.5%) with primary infertility and four patients (28.5%) with secondary infertility, resulting in 15 cases overall. Peritoneal factors were found in one patient (2.7%) with primary infertility and two patients (14.28%) with secondary infertility, adding up to three cases. Additionally, eight patients (22.2%) with primary infertility and one patient (7.14%) with secondary infertility had unexplained infertility, totaling nine cases. This distribution highlights that tubal and ovarian factors were the most common contributors to infertility, with primary infertility often linked to tubal issues and secondary infertility showing significant occurrences of both tubal and ovarian problems.

**Table 3:** Factors contributing to infertility in patients under study

Various factors	Primary infertility n(%)	Secondary infertility n(%)	Total
Uterine	4(11.1)	1(7.14)	5
Tubal	12(33.3)	6(42.85)	18
Ovarian	11(30.5)	4(28.5)	15
Peritoneal	1(2.7)	2(14.28)	3
Unexplained	8(22.2)	1(7.14)	9

## Discussion

In the present study involving 50 infertility cases, we found that 72% of patients had primary infertility, while 28% had secondary infertility. Deshpande et al. (2019) observed a similar pattern, noting a higher prevalence of primary infertility compared to secondary infertility [12]. Chanu et al. (2018) have reported that among 151 patients in their study, primary infertility was more common (58.28%) than secondary infertility (41.72%), with abnormal findings more prevalent in secondary infertility cases [10]. Notably, the most common age group for primary infertility in our study was 26-30 years (44.4%), whereas secondary infertility was predominantly seen in the 31-35 age group (50%). This age distribution underscores the impact of age on fertility and aligns with existing literature on reproductive health. Katole et al. (2019) and Nandedkar et al. (2014) have reported a similar trend in the age group corresponding to primary and secondary infertility [1,13]. 

The duration of infertility was predominantly one to five years for primary infertility cases, while half of the secondary infertility cases had experienced infertility for six to 10 years. This suggests that younger women may seek help earlier in their reproductive journey, while those with secondary infertility may face prolonged challenges before seeking treatment. However, several other factors act as barriers to pursuing infertility treatment. In a study conducted by Patra et al. (2022), that while most women seek infertility treatment, progression through treatment phases declines, often due to barriers like age, limited access, and financial constraints. Increased education and media exposure significantly boost the likelihood of opting for allopathic treatment [14].

Our findings indicate that tubal factors accounted for the majority of infertility cases (36%), with tubal blockage being the most common issue identified, while hydrosalpinx was observed in only a few cases. Our findings align with multiple previous studies, which have identified tubal factors as a leading cause of infertility in women, with incidence rates exceeding 30% in each study [15-17]. This is consistent with the understanding that tubal patency is critical for conception and highlights the importance of assessing tubal health in infertility evaluations. Ovarian factors contributed to 29.8% of infertility cases, with PCOS emerging as the most prevalent cause, followed by ovarian cysts and tubo-ovarian masses. These findings are consistent with previous studies showing that around 25% of infertility in women is caused due to ovarian factors, as reported in extensive reviews by Walker and Wyns (2024) and Carson and Kallen (2021) [3,9,18]. This reinforces the need for comprehensive hormonal evaluations in patients presenting with infertility. Additionally, our study identified a few cases of endometriosis (6%) and pelvic adhesions (5%), which can result in peritubal and omental adhesions, leading to distortion of pelvic anatomy. These findings are consistent with those reported by Godinjak and Idrizbegovic, emphasizing the role of such conditions in infertility [19].

Furthermore, DHL revealed uterine pathologies, including submucous fibroids, uterine anomalies, and intrauterine adhesions. These findings concur with previous studies attributing uterine causes to 10-15% of infertility cases [20-22]. DHL is particularly valuable in detecting uterine anomalies that may not be identified through ultrasound or other routine investigations. Chanu et al. (2018) also report that laproscopic assessments revealed more abnormalities than hysteroscopic evaluations, underscoring the effectiveness of DHL in identifying infertility issues [10]. Undetected uterine anomalies can lead to pregnancy loss, and surgical correction of these issues has been shown to dramatically improve pregnancy outcomes. The ability of DHL to facilitate the detection and removal of uterine polyps and fibroids further underscores its importance in enhancing fertility prospects.

Limitations

Although these findings contribute significantly to the existing literature, we acknowledge certain limitations as well. One limitation of the study is the relatively small sample size of 50 patients, which may limit the generalizability of the findings to a broader population. Additionally, the study was conducted at a single center, which may introduce a selection bias and limit the diversity of the patient population. Another limitation is the exclusion of patients with certain comorbidities or morbid obesity, which may affect the results since these conditions are common in cases of infertility. 

## Conclusions

Our study highlights the complex and multifactorial nature of infertility, with significant contributions from tubal pathologies, such as blockages, and ovarian disorders, including PCOS, as key contributors in our cohort. These findings underscore the need for thorough and precise evaluation of reproductive structures during infertility assessments. Advanced diagnostic tools like DHL were crucial in identifying underlying causes that may remain undetected through conventional methods like ultrasound or hormonal evaluations. DHL's ability to simultaneously evaluate the uterus, fallopian tubes, and ovaries offers a comprehensive approach to diagnosing mechanical and structural abnormalities contributing to infertility. Incorporating DHL into standard infertility workups can enable clinicians to provide more targeted and personalized treatment plans, potentially improving reproductive outcomes.
